# Impact of the SARS-COV-2 Pandemic Lockdown on Sexually Transmitted Urethritis in a Spanish Health Region

**DOI:** 10.7759/cureus.18921

**Published:** 2021-10-20

**Authors:** Oriol Yuguero Torres, Josep Fernandez, Elena Justribo, Eva González, Ana Vena

**Affiliations:** 1 Emergency Department, Universitat de Lleida, Lleida, ESP; 2 Dermatology, Institut de Recerca Biomèdica de Lleida (IRBLLEIDA), Lleida, ESP; 3 Primary Health, Institut Català de la Salut, Lleida, ESP; 4 Infectious Diseases, Institut de Recerca Biomèdica de Lleida (IRBLLEIDA), Lleida, ESP; 5 Geriatrics, Institut de Recerca Biomèdica de Lleida (IRBLLEIDA), Lleida, ESP

**Keywords:** std, emergencies, covid19, chlamydia, urethritis

## Abstract

Background

The incidence of sexually transmitted diseases (STD) has increased in recent years, especially in the young population. Urethritis is one of the most common clinical presentations of STD in emergency departments. During the SARS-COV-2 pandemic, in Spain lockdown lasted almost three months, and mobility was greatly restricted. This is the first study of these characteristics conducted in Spain.

Methods

A cross-sectional study of all patients treated for clinical symptoms of urethritis between March and June 2019 and between March and June 2020 was conducted. We evaluated patients’ sociodemographic and clinical variables.

Results

Seventy-nine patients were included in the study: 37 in 2019 and 38 in 2020 of whom 94.9% were men. The main symptoms were urethral discharge (59.5%) followed by dysuria (26.6%). Risky sexual relations were reported by 63.2% of patients in 2019, and this percentage decreased to 43.9% in 2020.

Conclusions

The number of patients attending an emergency department in our health region for urethritis did not undergo any variations between 2019 and 2020. No significant reduction in the number of cases of urethritis was observed, probably because people continued with unsafe sexual relations despite the social restrictions and difficulties posed by the lockdown.

## Introduction

The incidence of sexually transmitted diseases (STD) has increased in recent years, especially in the young population. *A study published this year showed an increase in the incidence of Chlamydia trachomatis infection among the youth* [1]*.* Prevention and control of these infections has been set as a public health priority. During the SARS-COV-2 pandemic in Spain, lockdown lasted almost three months, and mobility was greatly restricted. Every year 357 million new cases of the four most common types of STD (*C. trachomatis* (CT), *Neisseria gonorrhoeae* (NG), syphilis and *Trichomonas vaginalis* (TV)) are reported in subjects aged between 15 and 49 years. These epidemics have a particular impact on adolescent and young people’s health and life. Gonorrhea and *C. trachomatis* infection are major causes of infertility around the world [2].

At the end of the first wave of the COVID-19 pandemic, an Italian study [3] revealed that the pandemic did not halt sexual infections, and in their clinic, they reported nine cases of *C. trachomatis* infection. However, another study [4] in Cuba showed that cases of syphilis and gonorrhea decreased significantly during the period of social restrictions imposed due to the first wave of COVID-19. Events were similar in other countries [5].

In fact, concerns with the spread of STD during the COVID-19 pandemic and the consequences of their underdiagnosis led various public health professionals [6] to establish recommendations to control these infections.

Urethritis is one of the most common clinical presentations of STD in the emergency department [7]. Infections by NG and CT cause urethritis in male patients and cervicitis in women. The most common symptoms are urethral discharge and dysuria, which are classified as gonococcal urethritis and non-gonococcal urethritis, respectively [8].

## Materials and methods

Design

A cross-sectional study of all the patients treated for clinical symptoms of urethritis at the two main emergency departments of our health region between March and June 2019 and between March and June 2020 was conducted. Our health region, Lleida (Catalonia) has a reference population of 300,000 inhabitants.

We included all the patients above 16 years old who attended the ED during those periods. The *presenting symptoms* were genitorurinary symptoms, like dysuria, urethral discharge, or polyuria, and a urethritis was diagnosed.

Variables

We evaluated patients’ sociodemographic and clinical variables. Sociodemographic variables include age and gender. Regarding clinical variables, we studied symptom type and duration, the treatment received, and if we obtained a urethral discharge culture, the microbiological findings. If any microorganism is detected in the culture, a nucleic acid amplification test (NAAT) is carried out to detect other STDs just like *Ureaplasma urealyticum, Ureaplasma parvum, Mycoplasma genitalium, Trichomonas vaginalis, C. trachomatis, and N. gonorrhoeae*. Moreover, patients were asked about any risky sexual behavior (defining risky sexual behavior, as condomless sex with an unknown partner or under drugs effects) prior to symptoms.

Statistical analysis

For quantitative variables, we obtained the median of all the variables with a 95% confidence interval. Differences were studied using the chi-square test and Fisher’s exact test when the sample size was low. In both cases considering significant differences when p<0.05.

The project was evaluated and approved by the Research Ethics Committee of the Biomedical Research Institute of Lleida. Because it was a retrospective study, informed consent was not obtained.

## Results

Seventy-nine patients were included in the study: 37 in 2019 and 38 in 2020 of whom 94.9% were men. A general description of the sample is provided in Table 1. Patients’ main symptoms were urethral discharge (59.5%) followed by dysuria (26.6%).

**Table 1:** Description of the sample NA: not available; ^+^Other treatment includes anti-inflammatory treatments and antibiotics different from the above; p.overall shows the Mann-Whitney test p-value for quantitative variables (age and duration of symptoms) between 2019 and 2020. The Pearson’s Chi-squared test p-value is used to detect differences between 2019 and 2020 for qualitative variables, except for the distribution of gender, symptoms, and other STD detected, for which the Fisher’s exact test was used due to the presence of cells with very low frequencies. *Highlight the treatments with a frequency distribution significantly different between 2019 and 2020 according to the analysis of the adjusted standardized residuals. Thus, in 2020, ceftriaxone and azithromycin were significantly less used whereas other treatments were more frequently applied than in 2019. A Chi-squared test was used to detect differences between 2019 and 2020 samples.

	[ALL]	2019	2020	p.overall
Men, n(%)	75 (94.9%)	37 (97.4%)	38 (92.7%)	0.616
Women, n(%)	4 (5.06%)	1 (2.63%)	3 (7.32%)	
Age (median [min;max])	36.0 [16.0;88.0]	38.0 [21.0;88.0]	34.0 [16.0;76.0]	0.508
Symptoms				0.058
Urethral discharge, n(%)	47 (59.5%)	27 (71.1%)	20 (48.8%)	
Dysuria, n(%)	21 (26.6%)	7 (18.4%)	14 (34.1%)	
Pruritus, n(%)	7 (8.86%)	4 (10.5%)	3 (7.32%)	
Other, n(%)	4 (5.06%)	0 (0.00%)	4 (9.76%)	
Risky sexual relations	42 (53.2%)	24 (63.2%)	18 (43.9%)	0.137
Duration of symptoms (median [min;max])	3.00 [1.00;37.0]	3.00 [1.00;30.0]	3.00 [1.00;37.0]	0.766
Normal urine test, n(%)	58 (73.4%)	27 (71.1%)	31 (75.6%)	0.839
Treatment				0.027
Ceftriaxone and azithromycin, n(%)	38 (48.1%)	24 (63.2%)	14* (34.1%)	
Cefixime and azithromycin, n(%)	17 (21.5%)	7 (18.4%)	10 (24.4%)	
Other^+^, n(%)	24 (30.4%)	7 (18.4%)	17* (41.5%)	
Other STD detected, n(%)	2 (2.53%)	0 (0.00%)	2 (4.88%)	0.494
Need for repeat visit, n(%)	22 (27.8%)	8 (21.1%)	14 (34.1%)	0.296

Risky sexual relations were reported by 63.2% of patients in 2019, and this percentage decreased to 43.9% in 2020. However, this difference was not statistically significant.

In both years, patients attended the ED on the third day of symptoms manifesting. In 2019, the most common treatment was ceftriaxone/cefixime and azithromycin (81.6% of cases). In 2020, just 58.5% of patients received the recommended antibiotic treatment, the remainder being prescribed alternative, non-recommended antibiotic or analgesic treatment, and these differences were significant. Patients’ sexual partner was not reported by 84% of patients. Around one-fifth of patients (21.1%) returned to the ED after their first visit. This percentage increased to 34.1% in 2020.

In Table 2, we show characteristics of urethral discharge culture if available, since for 9 patients (23.7%) in 2019 and 13 patients (31.7%) in 2020 their culture was not requested.

**Table 2:** Urethral discharge culture characteristics ^+^Other organisms were identified: *Ureaplasma urealyticum*, *Ureaplasma parvum*, and *Trichomonas vaginalis*. p.overall shows the Chi-square test p-value for the urethral discharge culture and the Fisher’s exact test p-value for the distribution of germs among positive cultures (due to the presence of cells with very low frequencies). Both tests tell us if there are statistically significant differences between 2019 and 2020 outcomes. *Highlight the germs with a frequency distribution significantly different between 2019 and 2020 according to the analysis of the adjusted standardized residuals. Thus, in 2020 C. trachomatis showed a significantly higher frequency of cases whereas germs with very low frequency in 2019 (aggregated as “Other^+^”) showed no case in 2020.

	[ALL]	2019	2020	p.overall
Urethral discharge culture				0.05662
Sterile, n(%)	24 (30.4%)	8 (21.1%)	16 (39.0%)	
Positive, n(%)	33 (41.7%)	21(55.2%)	12 (29.2%)	
Distribution of germs among positive				
Chlamydia trachomatis, n(%)	8 (24.2%)	2 (9.5%)	6* (50.0%)	0.00286
Neisseria gonorrhoeae, n(%)	9 (27.3%)	8 (38.1%)	1 (8.3%)	
Mycoplasma genitalium, n(%)	9 (27.3%)	4 (19.0%)	5 (41.7%)	
Other^+^, n(%)	7 (21.2%)	7 (33.3%)	0* (0.0%)	

The urethral discharge culture was positive in 41.76% of cases, with no statistical differences (p=0.052). In 2020, there was an increase in CT-caused urethritis and a significant decrease in NG. These differences were statistically significant (p<0.05). The culture was not obtained for 31% of the patients seen in 2020 and empirical treatment was prescribed, due to the collapse of the laboratory due to COVID cases.

## Discussion

The number of patients who attended the ED presenting with urethritis in our health region did not undergo any variations between 2019 and 2020. This is the first study of these characteristics to be carried out in Spain. In addition, in Spain, the first wave required a severe lockdown with many social restrictions, and in our region, there was a second lockdown two months after the first cases were diagnosed.

The SARS-Cov2 pandemic had no effect on the incidence of this pathology in our ED. No statistical significance was observed in either the number of patients or their gender or age. *An important fact is that the number of women in our sample is really small. Probably because they used to attend to their primary care physicians and because the main cause of urinary tract infections among women are due to cystitis and specific symptoms of urethritis are less frequent. Moreover, we have few adolescent patients, because in our ED we attend patients above 16 years. Younger patients are attended in the pediatric section.*

No differences were observed in the length of time subjects wait before seeking medical attention. In both years, subjects attended the ED three days after the onset of symptoms. We believed this population would likely have delayed medical consultation due to the pandemic, but this was not the case. These results have been similar in countries with the lockdown of the same characteristics as Spain. In fact, in Australia, despite observing a drastic decrease in the period of confinement, it rapidly increased with the relaxation of the measures [9].

Interestingly, we did not observe a significant reduction in risky sexual relations. In a study carried out in other regions of Spain, it was observed that risk behaviors had not decreased despite mobility restrictions [10]. Moreover, only 15% of patients were able to identify their sexual partners to inform them that they may be infected with an STD. This is a worrying fact because it shows that risky behaviors are very difficult to control, and could explain the difficulty in containing the spread of STD.

Another interesting finding is that the treatment prescribed each year was significantly different. The most recommended treatments are ceftriaxone and azithromycin, as they are effective against gonococcal and non-gonococcal urethritis and reduce the risk of resistance [11]. In some cases, cefixime is also recommended [12]. Moreover, with such treatment, two basic principles in STD treatment are assured: single-dose treatment and medical supervision. In Catalonia, the resistance rate of NG to cephalosporins was lower than 5% while resistance to Ciprofloxacin is above 50% [13].

In 2019, emergency physicians usually chose appropriate treatment. However, antibiotics were not the main group of drugs prescribed in 2020. This is probably the main reason why subjects needed to return to the emergency department more frequently during that year. During the most restrictive period of the pandemic, the CDC recommended empirical treatments to try to reduce the risk of spreading the disease and the number of complications [14].

Moreover, in 2020, in more than 30% of cases, a urethral discharge culture was not obtained. As we said due to the busy days in the laboratory due to COVID cases, in those cases doctors prescribed empirical treatment without doing a culture. We have seen significant differences in the microorganism causing urethritis (p<0.05). NG and CT were the most predominant microorganisms observed. However, in 2020, CT showed a significantly higher frequency of cases. In fact, in the Tarin-Vicente study [10] in which all sexually transmitted diseases were analyzed, CT and NG infections continued to be the most prevalent. In Finland, they also observed no decrease in the notification of CT and NG cases during the lockdown [15]. However, in our sample, we observed that in 2019 the most prevalent microorganism was NG and in 2020 the most common was CT.

Our results differ from other studies that evaluated STD prevalence using the same methodology as ours [4,5]. In our case, we did not observe a significant reduction in cases of urethritis in our health region in spite of strict lockdown from mid-March to the end of May, probably due to the ease of access to emergency services. A study carried out in Catalonia observed a significant decrease in STD cases [16], but they attributed it to an underdiagnosis and to the difficulty of accessing a health center. In fact, in the United States, a very significant decrease was observed in 2020 compared to 2019 [17]. However, a very important rebound was subsequently observed. This suggests that cases did not decrease, but simply were not reported until the worst phase of the pandemic passed.

The main limitation of our study is its small sample. However, we believe its main strength is that in our health region, all patients with symptoms related to urethritis are required to attend to our emergency department and we have a truly representative sample. Another limitation is that in 2020 less urethral cultures were performed, and this can lead to a bias of missing data.

## Conclusions

During the strict lockdown, in our health region, we did not observe a significant reduction in cases of urethritis. People continued to have unsafe sexual relations despite the social restrictions and difficulties posed by lockdown, and we should be aware that the fight against STD during the COVID pandemic cannot and must not be overlooked.

Our study shows that despite the severe lockdown of the first wave of COVID, a significant decrease in urethritis cases was not observed in our region. There was not a decrease in sexual risky practices. Although other studies did show a significant reduction, this was attributed to underreporting rather than an actual reduction in cases. If despite the mobility restrictions and the fear of transmission (especially in the first months of COVID), the cases and risk relationships were maintained, this should make us propose new strategies for the control and containment of sexually transmitted diseases.
